# Empirical Models of Shear-Wave Radiation Pattern Derived from Large Datasets of Ground-Shaking Observations

**DOI:** 10.1038/s41598-018-37524-4

**Published:** 2019-01-30

**Authors:** Sreeram Reddy Kotha, Fabrice Cotton, Dino Bindi

**Affiliations:** 1 Helmholtz Centre Potsdam, GFZ German Research Centre for Geosciences, 14467 Potsdam, Germany; 2 University of Potsdam, Potsdam, Germany

## Abstract

Shear-waves are the most energetic body-waves radiated from an earthquake, and are responsible for the destruction of engineered structures. In both short-term emergency response and long-term risk forecasting of disaster-resilient built environment, it is critical to predict spatially accurate distribution of shear-wave amplitudes. Although decades’ old theory proposes a deterministic, highly anisotropic, four-lobed shear-wave radiation pattern, from lack of convincing evidence, most empirical ground-shaking prediction models settled for an oversimplified stochastic radiation pattern that is isotropic on average. Today, using the large datasets of uniformly processed seismograms from several strike, normal, reverse, and oblique-slip earthquakes across the globe, compiled specifically for engineering applications, we could reveal, quantify, and calibrate the frequency-, distance-, and style-of-faulting dependent transition of shear-wave radiation between a stochastic-isotropic and a deterministic-anisotropic phenomenon. Consequent recalibration of empirical ground-shaking models dramatically improved their predictions: with isodistant anisotropic variations of ±40%, and 8% reduction in uncertainty. The outcomes presented here can potentially trigger a reappraisal of several practical issues in engineering seismology, particularly in seismic ground-shaking studies and seismic hazard and risk assessment.

## Introduction

A seismic ground-shaking map predicts the spatial distribution of the shaking intensity in the nearby geographic area. Rapidly generated ground-shaking maps for a single event, such as the USGS ShakeMaps^1^, have become invaluable for public information, damage assessment, emergency responses, and engineering and scientific analyses. While ground-shaking maps for several thousand prospective earthquakes are required for probabilistic seismic hazard and risk assessment of spatially extended infrastructures. Such predictions combine the information on earthquake magnitude, geometry, and location with a ground-shaking prediction model, to estimate ground-shaking intensities (typically in terms of Pseudo-Spectral Acceleration, PSA) over a wide area around the epicenter. Often, empirical prediction models are developed for regions with sufficient ground-shaking data (e.g., recorded seismograms), while in regions with sufficient tectonic and geological information, physics based numerical simulations are preferred. Nevertheless, the practicality of seismic hazard and risk assessment is only as good as the underlying ground-shaking prediction model, especially in their description of the spatial variability of seismic wave amplitudes (in this study, PSAs).

S-waves, as the elastic shear-waves, are the strongest seismic (body) waves, and are responsible for shaking and damaging of man-made structures. Most empirical ground-shaking models are derived to predict the scaling of S-wave amplitudes with earthquake moment magnitude (*M*), and its distance from the affected site (*R*). Coupling the largest datasets^2,3^ and numerical simulations, recent ground-shaking prediction models^4,5^ have achieved considerable explanatory and predictive power. Despite being intuitive and computationally efficient, empirical ground-shaking models have one major limitation; these models assume and predict the radiation pattern of S-wave energy to be isotropic. This is far from reality, because a double-couple shear dislocation (e.g., an earthquake) radiates energy very differently from an explosive/implosive dislocation (e.g., a bomb).

The theoretical formulation^6^ of S-wave radiation patterns is anisotropic yet deterministic, and depends on the rupture geometry and focal mechanism. Empirically^7–10^ though, S-wave radiation patterns transition between deterministic and stochastic depending on the heterogeneity of propagation medium, which makes it difficult to calibrate and include in prediction models. Consequently, both numerical and empirical prediction models suffer from either miscalibration or oversimplification of the S-wave radiation phenomenon.

## Elusive S-Wave Radiation Patterns

A vertically dipping strike-slip earthquake in a homogenous half-space radiates S-wave energy as a four-lobed pattern: with the largest S-wave amplitudes in the rupture strike and normal directions (0^o^, ±90^o^, 180^o^), and relatively lower amplitudes elsewhere. Some empirical analyses^7–10^ have reported large isodistant azimuthal variations of S-wave amplitudes around the epicenters of various strong earthquakes, which suggested the need to include realistic radiation patterns into empirical^11^ and numerical prediction models. However, unlike their theoretical formulation, radiation patterns are observable only in a limited bandwidth of S-wave frequencies and in a limited epicentral distance range^12,13^. For some earthquakes^8,14^, the four-lobed S-wave radiation pattern was observed to be intact at frequencies as high as 16 Hz. While for others^7,15^, the four-lobes were completely disrupted by small-scale crustal heterogeneities^12,16^ beyond frequencies as low as 4 Hz. The mixing of SH–SV wave polarities during propagation in a layered crustal medium^15,17^ appears to distort the four-lobed pattern as well.

Regarding the frequency-dependence, S-wave radiation appears to transition from a deterministic to a stochastic process around frequencies of 1–4 Hz. Numerical simulations^17^ of wave propagation in randomized three-dimensional crustal velocity models have been able to reproduce this transitional range, but of course, the results are sensitive to the parametric description of crustal scattering properties (e.g., correlation length of medium heterogeneities). Based on observations and simulations, a few others^18,19^ elaborated and applied analytical models depicting a smooth, linear transition between anisotropic and isotropic radiation patterns in the frequency range 1–3 Hz, in order to study earthquake source and propagation effects.

In most studies related to S-wave radiation pattern, despite differences in underlying methodologies, the equally essential distance-dependence of radiation pattern is often ignored. Studies^20,21^ based on observed energy partitioning of S-waves revealed a very weak distance dependency for frequencies 2–5 Hz, and distance independent isotropic pattern beyond 6 Hz – but the dataset consisted of only three earthquakes recorded in the distance range 13–27 km. A more recent study^13^ based on observed spatial distribution of maximum amplitudes, used a larger dataset consisting of 13 strike-slip earthquakes (in Chugoku region, Japan) recorded in 0–150 km distance range. The resulting empirical model^13^ captures both frequency- and distance-dependence, wherein the cross-correlation coefficient between observed and theoretical low frequency S-wave (0.5–1 Hz) amplitudes decreases linearly (from 0.75) beyond a normalized hypocentral distance *log*(*kL*) ∼ 1.64, which is approximately 30 km when wave number *k* = 1.33 km^−1^. While a few earlier studies^12,22^ suggested that four-lobbed pattern remains intact up to 40 km, even for high frequencies (>4 Hz). Essentially, a good understanding, and robust empirical models of the frequency- and distance-dependent S-wave radiation pattern are sought.

Although there is a reasonable agreement across all these studies, we observe that most are based on rather small datasets consisting of few (predominantly) strike-slip events. In addition, we identify that: (1) the frequency range (e.g. 1–3 Hz) wherein the transition appears to occur, needs to evaluated, (2) the datasets were rather limited in terms of magnitude and distance ranges, and diversity of style-of-faulting (i.e. strike, reverse, normal, and oblique-slip events), and (3) most analyses were performed in the Fourier domain, which limits their adoption in engineering applications relying heavily on PSA (Pseudo-Spectral Acceleration) predictions^23,24^. In this study, we tackle these limitations using the recently published large datasets compiled of several thousand seismograms, from a variety of earthquake focal-mechanisms, occurring across the globe, and uniformly processed specifically for engineering applications i.e., code^23,24^ based design of earthquake resistant structures.

Even with such large datasets derived from spatially dense strong-motion networks in Japan^25^ and southern California, USA^26^, the imprint of S-wave radiation pattern remained untraceable and unquantifiable within the mixed-effects regression based empirical ground-shaking models^11^. Understandably, radiation pattern is a *secondary* physical effect masked by other dominant physical processes, including: geometric spreading and anelastic attenuation with distance, scaling with event magnitudes and event-to-event variability of rupture dynamics, and the strong influence of local soil conditions at a recording surface site. These difficulties explain why the well-known four-lobed S-wave radiation pattern has never been taken into account in the development of PSA predicting Ground-Motion Prediction Equations^11^. However, most surface sites with stiff layers of top-soil^23,24^ and the overlying man-made structures^27^ resonate with a fundamental frequency from 0.2 Hz to 5 Hz, which coincides with the frequency range where the anisotropy of S-wave energy distribution is most prominent^28^. Therefore, it is critical that the spatial anisotropy of S-wave radiation pattern is incorporated into Ground-Motion Prediction Equations^11^ – the fundamental components of Probabilistic Seismic Hazard Assessments and rapidly deployable ground-shaking maps.

## Empirical Evidence of S-Wave Radiation Patterns

In our recent studies^29,30^, we tackled the various assumptions and limitations in development of mixed-effects regression based empirical ground-shaking models. Taking advantage of recently published large datasets^2,3,31^ and mixed-effects regression analyses^32^, we systematically quantified and isolated the dominant physical processes that control the spatial variability of ground-shaking. In the present study, we have gone a step further to analyze the *left-over* regression residuals, those quantifying the record-to-record variability of observed ground-shaking, from two empirical ground-shaking models^33,34^ (equation 1) developed from two recently compiled datasets^2,3^ of active shallow crustal earthquake recordings: one^3^ from the Japanese KiK-net^25^ strong-motion network; and another as a global NGA-West2 dataset^2^ compiled mostly of events from southern California^26^ (USA), plus a few large events from Italy, Taiwan, Turkey, China, Alaska, Georgia, Montenegro, New Zealand, and other active seismic regions.

1 $$\mathrm{ln}(PSA)={f}_{R}(M,R)+{f}_{M}(M)+\delta {B}_{e}+\delta S2{S}_{s}+\delta W{S}_{e,s}$$

The most common expression of S-wave amplitudes used in engineering seismology and earthquake engineering is an average^35^ of two-component (i.e., NS, EW) horizontal PSAs, for a range of spectral periods (e.g. T = 0.01–10.0 s). All of the ground-shaking datasets^2,3^ disseminate the observed ground-shaking intensity data only in terms of PSAs. The two ground-shaking models^33,34^ we considered were also developed to predict PSAs, because most seismic design codes^23,24^ rely on predicted PSAs. PSAs are essentially the peak acceleration responses to seismic excitation (i.e., a seismogram) of a suite of viscous damped (5% or other) single-degree-of-freedom oscillators with fundamental frequencies ${f}_{osc}=\frac{1}{T}$. It is true that conversion of ‘*as recorded*’ Fourier amplitudes to ‘*engineering purposed*’ PSAs is nonlinear^36^, which often makes the physical interpretations made in either of the domains non-interchangeable. However, within the earthquake moment magnitude (*M*3.4–*M*6.9) and period range of interest in this study (*T* = 0.1–1.0 s), responses of a single-degree-of-freedom (i.e., the PSAs) are dominated by the Fourier amplitudes of S-wave frequencies around f_osc_^36^. Hence, we can interpret the radiation pattern effects in PSA residuals as Fourier spectral features of the seismic input.

Typically, empirical ground-shaking prediction models (e.g., equation 1) are regressed from datasets of observed PSAs at several surface sites from multiple past earthquakes in a region. In equation (1), the fixed-effect *f*_*R*_ (*M*, *R*) captures the geometric spreading and apparent anelastic attenuation of PSAs with rupture moment magnitude (*M*) and distance to site (*R*), and *f*_*M*_ (*M*) captures the scaling of PSAs with *M*. *δB*_*e*_ and *δS*2*S*_s_ are the random-effects quantifying event-to-event (event index *e*) and site-to-site (surface site index *s*) variabilities, respectively. The *left-over* record-specific residuals *δWS*_*e,s*_ (of event *e* recorded at surface site *s*) contain all of the phenomena that evade the fixed- and random-effects, including the four-lobed S-wave radiation pattern. For brevity, we refer interested readers to the articles^33,34^ elaborating on the ground-shaking model development.

**Figure 1:**
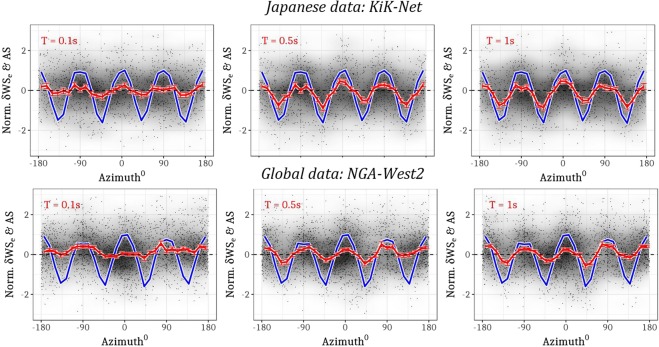
Empirical evidence of S-wave radiation patterns in residuals from records at R ≤ 100 km for strike-slip events in Japan: KiK-net (top) and Global: NGA-West2 (bottom) datasets. Panels show normalized residual *δWS*_*e,s*_ (black) and binned mean of normalized *δWS*_*e,s*_ (red) extracted from mixed-effects regression of PSAs with T = 0.1 s, 0.5 s, 1 s (left to right), and binned means of normalized, theoretical far-field S-wave amplitudes (blue) corresponding to each record.

Figure 1 presents the empirical evidence of S-wave radiation patterns in ground-shaking data (i.e., residuals *δWS*_*e,s*_). While the most recent studies^13,22^ considered 13 events, we select 1615 and 3193 *δWS*_*e,s*_ residuals (records) at *R* ≤ 100 km of 91 and 78 strike-slip events from the KiK-net^3^ and NGA-West2^2^ datasets, respectively. These residuals are standardized i.e., divided by their standard deviation (*ϕ*_0_), and plotted against the azimuth of the recording stations measured with respect to the rupture strike direction. As no single event is recorded by a complete 360° and 0 km to 100 km network, by re-orienting the 142 and 299 surface site locations (of KiK-net and NGA-West2, respectively) to the rupture strike as datum, the radiation pattern can be seen as an *average* trend over several dozen earthquakes of size M3.0–M6.0 (Fig. 1, red curve). The four-lobed radiation pattern of a vertical dipping strike-slip event produces greater than average ground-shaking (over all azimuths) in the strike parallel and normal directions (azimuth = 0°, ±90°, ±180°), and the weakest ground-shaking in between (azimuth = ±45°, ±135°). The red curve in Fig. 1 represents the binned means of the standardized *δWS*_*e,s*_ residuals, and shows a clear trend that is characteristic of the expected four-lobed radiation pattern.

An important point regarding the data selection is that, we did not specifically identify and remove the records (and corresponding *δWS*_*e,s*_) with possible directivity effects. A recent study^37^ showed that a few small-moderate sized earthquakes of *M*_*w*_ ≤ 5.7, which constitute 90% of the data we used, may show directivity effects. However, the azimuthal variation of rupture directivity effects is gradual over the ±90^0^ range in the strike-parallel directions (0^0^ and 180^0^), while that from radiation pattern is more rapid within ±45^0^ range along strike-parallel (0^0^ and 180^0^) and perpendicular directions (−90^0^ and 90^0^). The empirical radiation pattern we show in Fig. 1, averaged over several strike-slip events, could be effected by rupture directivity of a few events. Although not discussed here, such events are flagged as those deviating significantly from the radiation pattern, and can be investigated for possible near-source effects.

For reference, we overlay the theoretical far-field S-wave radiation pattern $(AS=\,\sqrt{{F}_{SH}^{2}+{F}_{SV}^{2}})$, zero-centered and standardized, as the blue curve in Fig. 1. The theoretical far-field S-wave amplitudes for point-source dislocation in a homogenous half-space, *F*_*SH*_ and *F*_*SV*_, are estimated for each record using the Aki and Richards^6^ formulae. These formulae require parametric information on the rupture focal mechanism^2,3^ (i.e., strike, dip, rake, hypocentral depth), crustal S-wave velocity model to calculate the take-off angles, epicentral distance, and azimuth of the recording station with respect to event epicentre^2,3^. Instead of an actual crustal velocity model, we used a take-off angle table^38^ provided by the Japanese Meteorological Agency. Although *AS* (as with $\sqrt{{F}_{SH}^{2}+{F}_{SV}^{2}}$) are all positive values, these were zero-centered and standardized for visualization in Fig. 1. Clearly, the theoretical and empirical amplitudes show identical azimuthal dependence.

**Figure 2:**
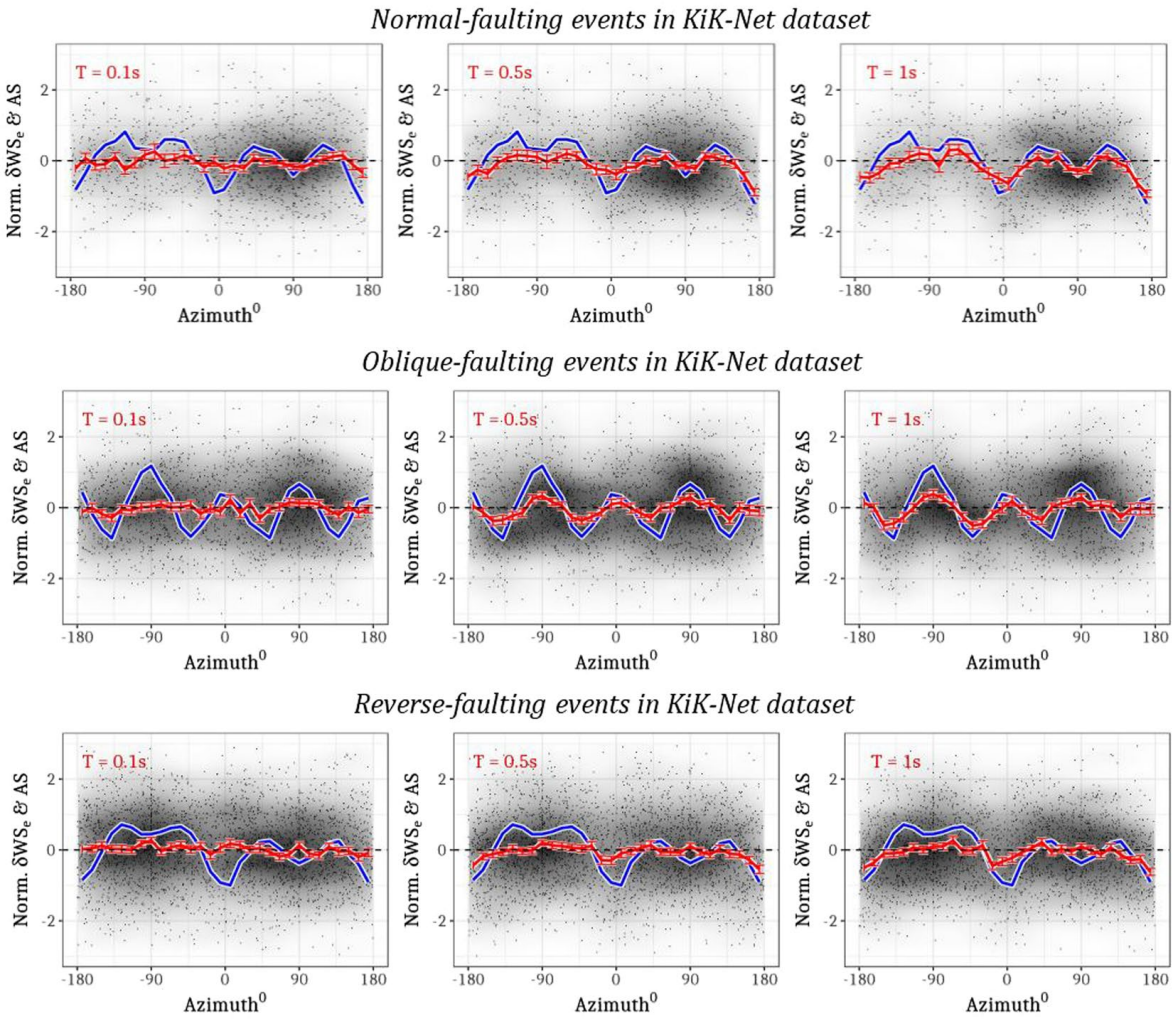
Empirical evidence of S-wave radiation patterns in residuals from records at R ≤100 km of normal, oblique, and reverse-slip events (top to bottom) from the Japanese KiK-net dataset. The panels show the normalized residuals *δWS*_*e,s*_ (black), binned means of normalized residuals *δWS*_*e,s*_ (red), and binned means of normalized, theoretical S-wave amplitudes (blue) that correspond to each record (residual) for T = 0.1 s, 0.5 s, 1.0 s (left to right).

**Figure 3:**
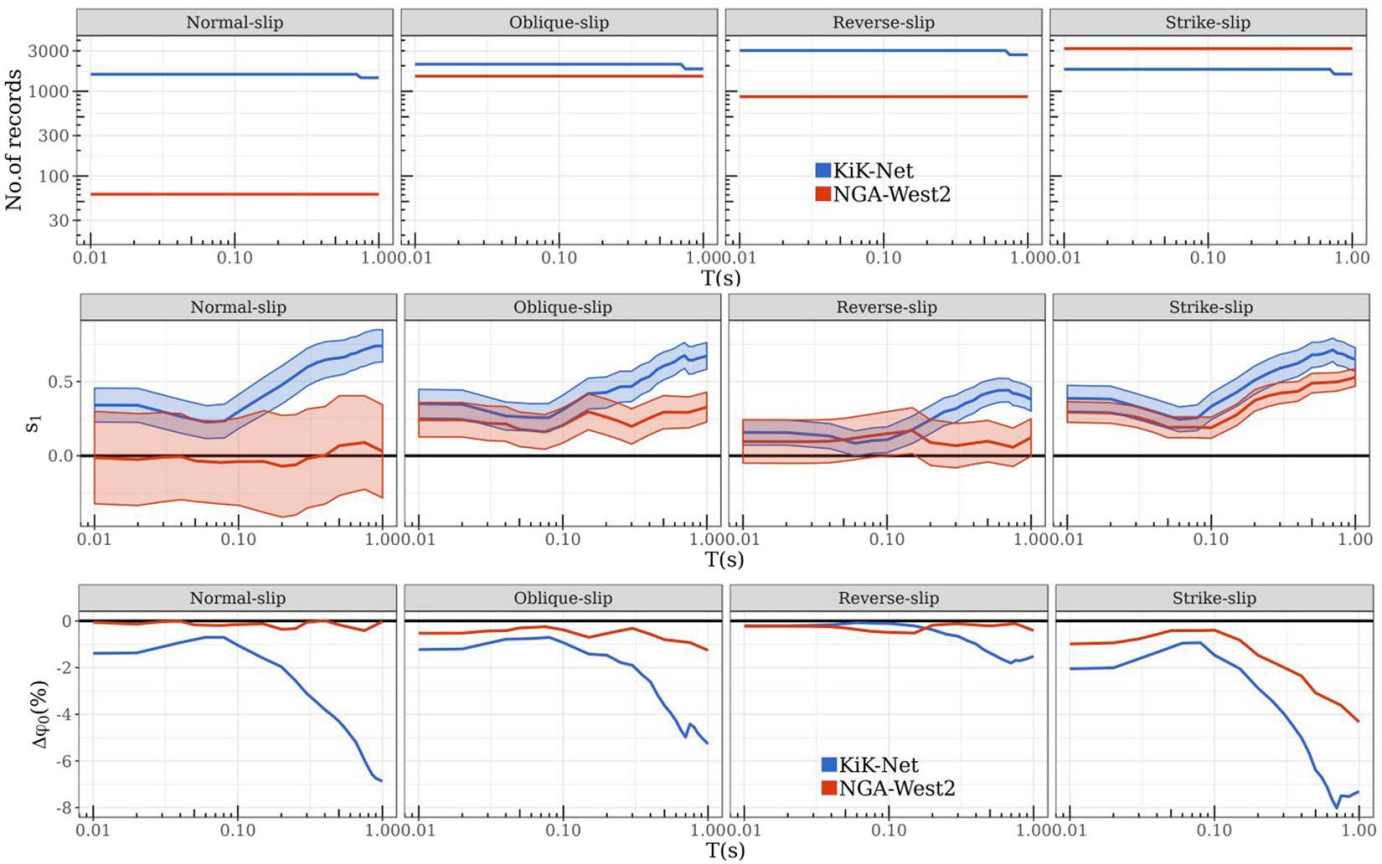
Calibration of style-of-faulting and period-dependent (T = 0.01 s–1.0 s) linear relationships between theoretical far-field S-wave radiation amplitudes (AS) and aleatoric residuals (*δWS*_*e,s*_). Top panels: the (decreasing) number of records with increasing T at R ≤ 100 km of oblique, normal, reverse, and strike-slip events in the KiK-net (blue) and NGA-West2 (red) datasets. Middle panels: the estimated period-dependent s_1_ (with 95% confidence interval) of equation (2), which quantifies the presence of S-wave radiation patterns in ground-shaking residuals. Bottom panels: the reduction in the ground-shaking variability (ϕ_0_) with the removal of the S-wave radiation pattern through the linear relationship.

While Fig. 1 shows the evidence of S-wave radiation patterns for strike-slip events from both the KiK-net and NGA-West2 datasets, Fig. 2 presents similar plots for normal (1468 records from 64 events in M3.5–M6.0), reverse (2747 records from 198 events in M3.5–M6.0), and oblique (1864 records from 102 events of M3.5–M5.5) faulting events in the KiK-net dataset (including six events larger than M6.0). Since the global NGA-West2 dataset contains only large magnitude normal and reverse faults from diverse tectonic regions across the world, the coherency between theoretical and observed radiation patterns is not as clear as with the numerous smaller events of the Japanese KiK-net dataset (see Fig. 3 top-panel for data distribution).

The theoretical far-field S-wave radiation pattern, formulated for a point-source dislocation in homogenous, isotropic half-space, is deterministic and frequency-independent. Of course, these formulae cannot be used to model high frequency S-wave radiation pattern, where the complex crustal heterogeneities no longer qualify the propagation medium as a homogenous, isotropic half-space. We treat the theoretical pattern only as a *scale* to measure the transition of observed radiation between isotropic and anisotropic patterns. In this setup, the mismatch between the theoretical (blue lines in Figs 1 and 2) and empirical radiation patterns (red lines Figs 1 and 2) is maximum at short period PSA (Figs 1 and 2, *T* = 0.1 s), which are analogues to high-frequency S-wave amplitudes. Moving towards longer periods (Figs 1 and 2, *T* = 0.5 and 1.0 s), the agreement between the empirical and theoretical patterns improves, which indicates the characteristic transition from stochastic to deterministic phenomenon. Despite using data from scores of earthquakes across Japan and southern California, where crustal structures are known to be highly complex and heterogeneous, the theoretical idealizations appear to work surprisingly well – at least for low S-wave frequencies.

## Transition from Stochastic to Deterministic Phenomenon

Following the non-parametric analyses, we derived parametric models aiming to extract the S-wave radiation anisotropy from the ground-shaking residuals, and reintroduce anisotropic adjustments into the isotropic prediction models. For each dataset^2,3^ independently, using a linear mixed-effects regression algorithm (lmer)^32^, we fit a simple, linear relation between the theoretical far-field S-wave radiation amplitudes and the residuals from isotropic ground-shaking models^33,34^. In equation (2), *s*_0_ is the intercept and *s*_1_ is the slope. Both *s*_0_ and *s*_1_ are set to vary with style-of-faulting (i.e., oblique, normal, reverse, strike-slip) in the mixed-effects regressions.

2 $$\delta W{S}_{e,s}={s}_{0}+{s}_{1}\,.\,A{S}_{e,s}$$

The top panels of Fig. 3 show the number of residuals/record used in deriving the *s*_1_ (and *s*_0_) values for the range *T* = 0.01 s to 1.0 s for each style-of-faulting and dataset. Most of the NGA-West2 data is constituted by strike-slip and oblique-slip events, while the distribution is more even in KiK-net dataset – the style-of-faulting dependent values of *s*_1_ (and *s*_0_), estimated as random-effects in an lmer^32^ of equation (2), is to account for such dataset imbalances.

There is a reason we limit our analyses to the period range T = 0.01–1 s. The KiK-net strong-motion dataset^3^ we used is the product of an automatic processing of ~150,000 seismograms from the NIED KiK-net database^25^, and is specifically built for engineering ground-shaking (PSA) studies such as ours^33^. Only through such automatic processing algorithms, it is possible to compile large datasets with thousands of records. However, the limitation of automatic processing is that we are required to apply a strict high-pass frequency filtering^39^ criterion to discard records with limited usable low-frequency content. Consequently, the number of usable KiK-net^3^ records drops rapidly for periods longer than T = 1 s. The NGA-West2 dataset^2^ on the other hand, is a product of manual processing of seismograms from active shallow crustal earthquakes, and therefore allows a wider usable period range of T = 0.01–10 s. However, to maintain consistency across datasets, we limited our investigation only in the period range T = 0.01–1 s. Further details on data selection are available in the relevant articles elaborating on the development of Ground-Motion Prediction Equations^33,34^ that we used in this study.

### Frequency Dependence of Radiation Pattern

The agreement between theoretical and empirical radiation patterns is period (*T*) dependent. In the middle panels of Fig. 3, considering *s*_1_ as a measure of correlation between empirical and theoretical patterns, and approximating f_osc_ (1/*T*) as S-wave frequencies, the monotonically increasing trend of *s*_1_ from *T* = 0.1 s to 1.0 s (f_osc_ = 1–10 Hz) can be interpreted as the transition of the S-wave radiation pattern from a stochastic to a deterministic process, which supports earlier findings^17^. Strike-slip events (Fig. 3, middle-right panel) from both datasets exhibit best the transition regime of the S-wave radiation patterns in their residuals – with statistically significant (as shown by its 95% confidence interval) estimates of *s*_1_. Normal, oblique, and reverse-slip events of the KiK-net show a clear trend as well, while there is very little data from normal-slip events in NGA-West2.

The efficiency of equation (2) in capturing and removing S-wave radiation anisotropy from the *δWS*_*e,s*_ is measured in terms of the reduction in prediction variance (*ϕ*_0_, the standard deviation of *δWS*_*e,s*_) of the parent ground-shaking models^33,34^. In the bottom panels of Fig. 3, we see up to 8% reduction in ϕ_0_ for normal, oblique, and strike-slip events of the KiK-net dataset. Such reductions in the ground-shaking prediction variance are highly sought in Probabilistic Seismic Hazard Assessment^40^. Reverse-slip events do not show as much reduction – we discuss this in the following section.

### Distance Dependence of Radiation Pattern

A recent study^41^ asserted that, for large earthquakes, radiation pattern controls the near-source saturation of ground-shaking. A few earlier studies^12,13,22^, based on small to moderate sized earthquakes, observed that the four-lobbed radiation pattern remains intact up to 40 km, beyond which seismic wave scattering and diffraction in the heterogeneous crust distorts the radiation pattern^42^. While these studies were based on a few strike-slip events in a locality, we extend the investigation with several more strike, normal, reverse, and oblique-slip events all over Japan.

**Figure 4:**
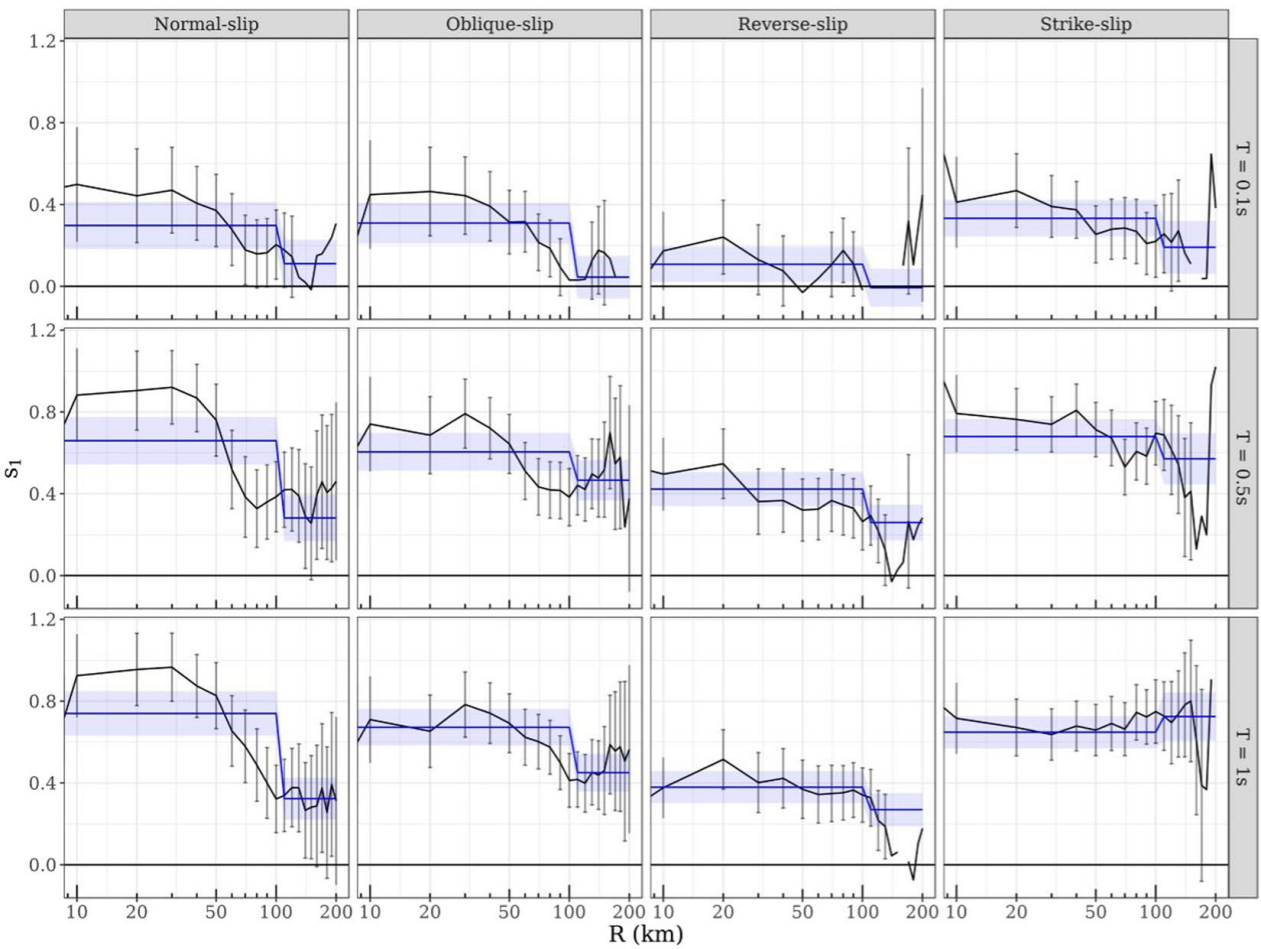
Distance dependence of radiation pattern for normal, oblique, reverse, and strike-slip events in the KiK-net dataset at T = 0.1, 0.5 and 1 s: Correlation between the theoretical and empirical radiation patterns is measured in terms of s_**1**_ in equation (2), for distance bins 0–30 km, 10–40 km, 20–50 km and so on. The solid black line and error bars depict bin-wise s_**1**_ and 95% confidence estimates respectively. The solid blue line and error ribbon depicts the s_**1**_ and its 95% confidence interval estimated for distance bins R ≤ 100 km and R > 100 km. Note that the blue lines assume values shown in middle panel of Fig. 3. Evidently the period and distance-dependence of S-wave radiation pattern is also dependent on the style-of-faulting of the events. While the strike-slip events show good correlation with theoretical expectation for epicentral distances larger than 100 km as well for T = 1s, normal and oblique-slip events show a much earlier decay starting at R ~ 40 km.

In Fig. 3, the frequency-dependence of radiation pattern for each style-of-faulting is estimated and presented using recordings with R ≤ 100 *km*. While Fig. 4 presents the distance-dependence of radiation pattern within this 100 km range, and up to 200 km, for normal, oblique, reverse, and strike-slip events at T = 0.1, 0.5 and 1 s. To demonstrate the distance-dependence, we estimate the *s*_1_ (equation 2) within each moving distance window of 0–30 km, 10–40 km, 20–50 km, and so on up to 200 km. For brevity, we present the results only for the KiK-net dataset.

Figure 4 shows that the period- and distance-dependence of S-wave radiation pattern is strongly dependent on the style-of-faulting of the events. Firstly, for strike-slip events, the *s*_1_ values at T = 0.5 s are significant (95% confidence intervals) up to 100 km. For long period PSAs (T = 1 s), which we consider comparable to low frequency S-wave amplitudes, we observe that significant *s*_1_ estimates persist up to 200 km. On the other hand, normal and oblique-slip events show a much earlier decay in correlation starting at R ∼ 20–40 *km*, depending on the frequency.

Reverse-slip events are peculiar in a few aspects. For instance, despite being the largest contributors to the KiK-net dataset in terms of number of events and records, they show a weaker correlation (smaller *s*_1_ in Fig. 3) values, and lesser reduction in aleatory variability, than other style-of-faulting events. At the same time, the distance-dependent decay (for T = 1 s in Fig. 4) starts to be significant at around 100 km – much later than normal and oblique-slip events. Empirical^43,44^ and simulation^45^ based studies showed that the hanging-wall effects of reverse-slip events may persist up to 100 km, and are sensitive to magnitude, dip, and depth to top-of-rupture plane. Observations from M7.6 Chi-Chi (Taiwan), M6.7, Northridge (New-Zealand), M6.3, L’Aquila (Italy), and M7.9, Wenchuan (China) support these simulations^45^; wherein, equidistant ground-shaking intensities (PSAs) on the hanging-wall could be three times larger than those on the foot-wall side of the rupture. Such large variability in observed ground-shaking due to effects finite-fault geometry (trapping of waves in the hanging-wall *wedge*), along with crustal heterogeneity (from crustal fractures), warrants a more detailed region-dependent investigation of reverse-slip events.

Based on Fig. 4, we suggest that the distance-dependence of S-wave radiation pattern integrity is also dependent on the focal-mechanism and spectral period (T), and vice-versa. While earlier studies suggested the radiation pattern is intact only up to 40 km, we find that for strike-slip events the range could be much longer, especially for long period PSAs (low frequency S-wave amplitudes). It is important to note that, focal mechanisms are characterized by tectonic environments, such as maximum shear stress orientation^46^. The distance dependence we observe to be characteristic of focal mechanisms, could as well be related to regional differences (within Japan)^47^ in scattering and absorption^12,39^. With this in mind, we intend a more thorough investigation of the magnitude, depth and crustal structure dependencies of s_1_. For interested readers, all the data plotted in Figs 1–4 are provided as electronic supplements for further investigations.

## Anisotropic Ground-Shaking Predictions

**Figure 5:**
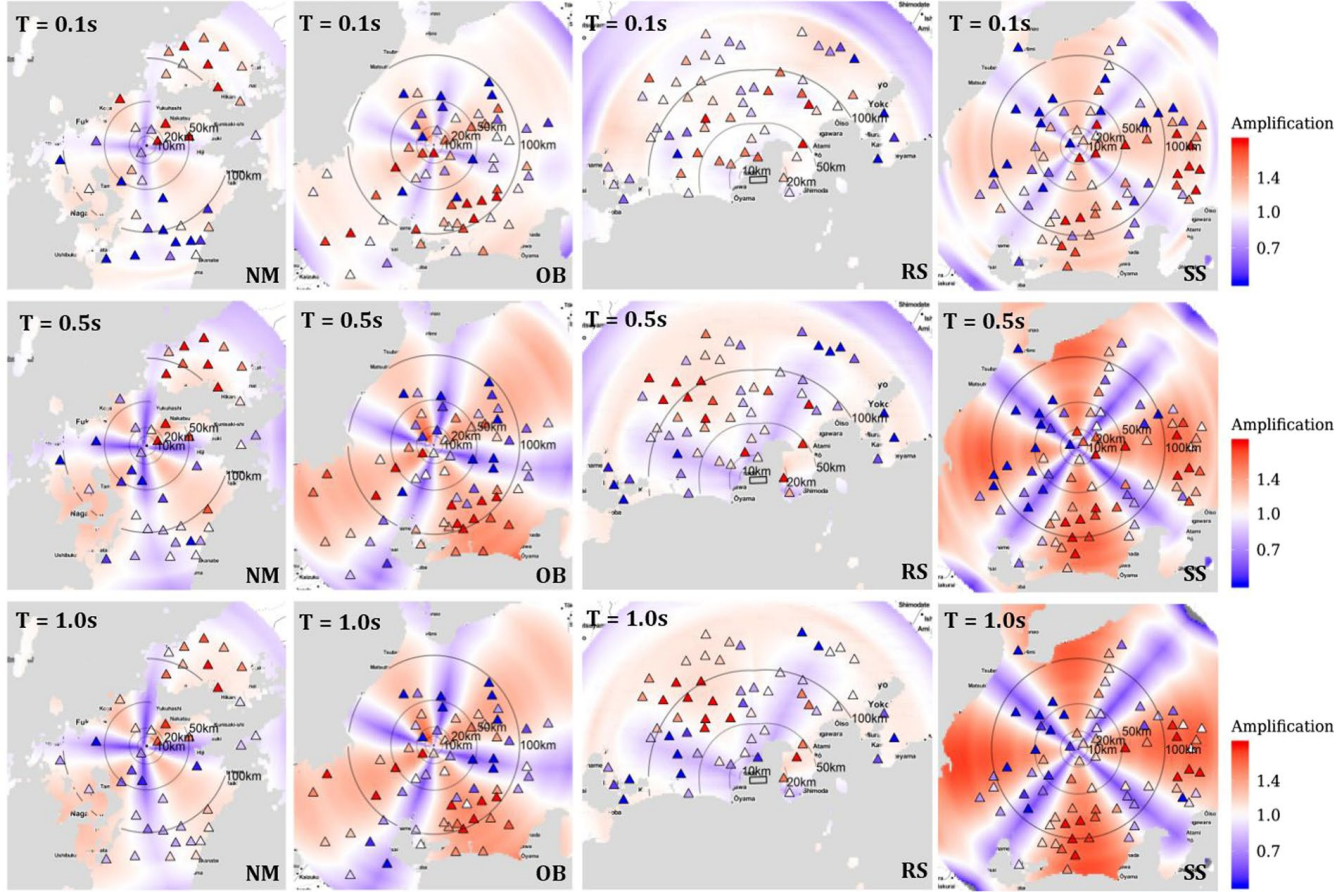
Period-, distance- and style-of-faulting dependent anisotropic ground-shaking amplification predictions for four events in Japan (NM: Normal, OB: Oblique, RS: Reverse, and SS: Strike-slip from left to right panels), for periods T = 0.1, 0.5 and 1 s (top-to-bottom panels). Each panel shows the R ≤ 200 km region (with 10, 20, 50,100 km contour lines) around the rupture trace, with color coding to reflect anisotropic increases (red)/decreases (blue) in ground-shaking with respect to the isotropic predictions, as a result of including the S-wave radiation pattern in empirical ground-shaking predictions. Overlying markers indicate the locations of recording sites, which are also color coded to reflect systematically higher (red) or lower (blue) than median isotropic ground-shaking model predictions. Wherever the background colors coincide with marker colors, the prediction model captures the observed anisotropic spatial variability of the ground-shaking reasonably well.

For a new event, given its rupture focal-mechanism and regional S-wave crustal velocity structure (or take-off angle table)^38^, the theoretical S-wave radiation amplitudes (*AS*) in its vicinity can be estimated, which can then be translated into anisotropic ground-shaking adjustments compatible with the isotropic ground-shaking prediction models^33,34^. We use the period-, distance- and style-of-faulting dependent coefficients *s*_0_ and *s*_1_ derived from the Japanese KiK-net dataset^3^ (shown in Fig. 4) to predict the radiation patterns of four well-recorded events available in the dataset (Table 1). Instead of comparing observed and predicted PSAs for each event at *T* = 0.1, 0.5 and 1 s, Fig. 5 compares the predicted anisotropic amplification/attenuation (equation 2 with coefficients in Fig. 4) against the observed deviation (i.e., residual *δWS*_*e,s*_ at each surface site *s* for event *e*) from isotropic, event- and site-specific PSA predictions by the KiK-net based ground-shaking model^33^. Generic, event- and site-independent isotropic PSA predictions can be estimated using only the fixed-effects of equation (1), whose regression coefficients are available as an online resource^33^. These generic predictions can then be adjusted to event- and site-specific predictions^30^ using the random-effects (is *δB*_*e*_ and δ*S*2*S*_*s*_) provided in the electronic supplements of this article, which also contain the residuals (*δWS*_*e,s*_).

**Table 1:** Event metadata of the four events shown in Fig. 5.

Event	Style-of-faulting	Focal-mechanism (dip, rake, strike)	Location (WGS84)	Origin (JST)	M_W_	Depth (km)
NM	Normal	(54°, −120°, 69°)	(130.89°E, 33.37°N)	25/06/2009 23:04	4.5	8
OB	Oblique	(88°, 35°, 332°)	(137.11°E, 35.76°N)	27/07/2004 00:55	4.2	8
RS	Reverse	(51°, 63°, 88°)	(138.50°E, 34.79°N)	11/08/2009 05:07	6.2	20
SS	Strike	(90°, −175°, 85°)	(137.70°E, 35.91°N)	13/06/2008 11:22	4.3	20

We choose four events with more than 30 recordings at *R* ≤ 200 km, and predict the S-wave radiation pattern in a 200 km radius around the rupture epicenter. The event metadata for the four events is provided in Table 1, while the event- and site-specific random-effects are provided in the electronic supplements. The colors in Fig. 5 reflect anisotropic increases (red) and decreases (blue) in the ground-shaking with respect to the isotropic event- and site-specific predictions in the region. The predicted radiation patterns are overlain by the locations (markers) of the recording surface sites, which are color coded for observed ${e}^{\delta W{S}_{e,s}}$, to indicate larger or smaller than predicted isotropic event- and site-specific ground-shaking. Essentially, we compare the anisotropic amplifications from an *average* model derived over several events in the Japanese KiK-net data with the anisotropic observations for well-recorded events, and this appears to work quite well. Qualitatively, the anisotropic ground-shaking predictions capture the observed anisotropy very well, which is of course period- and distance- dependent for each style-of-faulting. Quantitatively though, where the calibrated model suggests S-wave intensities that are 0.7-fold to 1.4-fold the median isotropic event- and site-specific ground-shaking predictions, the observations $({e}^{\delta W{S}_{e,s}})$ show much stronger anisotropy of the order of 0.5-fold to 2-fold. Note that our models capture the ‘*average*’ S-wave radiation pattern over several sparsely recorded *M*3.4 to *M*6.9 events that are scattered across Japan. This means that some events (in some regions of Japan) show stronger correlations between the theoretical S-wave radiation patterns and the observed anisotropy of the ground-shaking, while others fall short. In addition, near-source effects such as the directivity and hanging-wall effects, crustal heterogeneities, and complex 2D/3D site-responses could be exaggerating the mismatch between the observations and predictions.

Figures 1 and 2 show a very weak correspondence between theoretical and observed radiation patterns at T = 0.1 s, while Figs 3 and 4 show that the *s*_1_ values are either small or insignificant at T = 0.1 s. Consequently, our predictions in the top-panels of Fig. 5, corresponding to T = 0.1 s, produce relatively weak anisotropy, as indicated by the fainter colors (white meaning no anisotropy). Not to mention, theoretical far-field S-wave radiation equations cannot be applied at short periods (T = 0.1 s), where the assumptions of crustal homogeneity and isotropy do not hold anymore.

## Implications in Seismic Ground-Shaking Studies

S-wave radiation pattern is an extensively studied phenomenon in seismology. They have been repeatedly observed and quantified from observed S-wave amplitudes of a few well-recorded strike-slip events in the Fourier domain, or sometimes Peak Ground Acceleration and Velocity. However, within engineering seismology, they have never been quantified over large datasets of PSAs (Pseudo-Spectral Accelerations) from several normal, oblique, reverse, and strike-slip events – PSAs are the standard ground-shaking intensity measures applied in structural design codes, probabilistic seismic hazard and risk assessments, and ShakeMaps. The reasons for this include the limited spatial density of strong-motion networks to observe the patterns for any single event, and the dominance of other ground-shaking attenuation processes. In this study, we have revealed the imprint of S-wave radiation pattern in the regression residuals of existing, isotropic ground-shaking prediction models.

The novelty of our approach is to systematically remove the dominant physical effects from the recorded ground-shaking data, and to reorient the strong-motion network to emulate a complete azimuthal (0–360°) and distance coverage (0–200 km). The S-wave radiation pattern becomes evident, with its characteristic transition from a stochastic at high frequencies (short period PSAs) to a deterministic at low frequencies (long period PSAs) phenomenon. The transition appears to depend also on the events’ style-of-faulting and distance to the recording surface sites. We calibrate a period-, distance- and style-of-faulting dependent empirical model (equation 2) correlating theoretical far-field radiation coefficients with the empirical ground-shaking model residuals.

Our empirical radiation pattern models are simple and practical. The coefficients *s*_0_ and *s*_1_ of equation (2), derived from the KiK-net and NGA-West2 datasets, can be applied to the respective isotropic ground-shaking prediction models^33,34^ as in: $\mathrm{log}(PS{A}_{anisotropic})=\,\mathrm{log}(PS{A}_{isotropic})+{s}_{0}+{s}_{1}.AS$. These adjustment factors have an impact on ground-shaking maps^1^; with at least ±40% change in the isodistant predictions, depending on the site azimuth and distance relative to the event epicentre. In complement, the prediction variance ϕ_0_ is reduced by up to 8% for the KiK-net dataset based ground-shaking model.

Regional dependence^39,46,47^ of adjustment factors (*s*_0_ and *s*_1_ of equation 2) is an issue worth investigating, given that we do observe some event-to-event (or regional) variability - but that would require a more complex treatment of crustal heterogeneities. Our method, producing average adjustment factors, aims to balance model complexity and ease of applicability, so that anisotropic adjustment factors can be estimated for any empirical ground-shaking prediction model.

Our study intends a new approach to the inclusion of style-of-faulting effects in empirical ground-shaking prediction models. For instance, several ground-shaking models^4,5^ derived from the NGA-West2^2^ and RESORCE^31^ datasets (used in USGS^48^ and SHARE^49^ hazard maps) propose average isotropic, style-of-faulting and frequency-dependent adjustment factors. These factors were shown to vary considerably from one model to another and were not representative of any clear physical phenomenon^50^. While some studies could not constrain these adjustment factors^29,51^, others could only derive them for large earthquakes^4^. We demonstrate here that the lack of robustness in handling the style-of-faulting is in-fact from the assumption of averaged distance-independent isotropy, where distance and style-of-faulting dependent anisotropy appears to be prevalent.

From the lack of detailed knowledge of wave-propagation and rupture processes, physics-based broadband ground-shaking simulations^52^ usually consider isotropic radiation patterns that arise from the average of the theoretical radiation patterns over a suitable range of azimuth and take-off angles^53^ for high-frequency S-waves. Some recent developments have reproduced the homogenization of radiation pattern effects at higher frequencies using heterogeneous short-length scaled-rupture mechanisms^54^ or deterministic numerical modeling in three-dimensional heterogeneous Earth crust models. The empirical evidence presented in this study can be used to calibrate these simulations and the parameters that describe the medium-scattering properties and small-scale random-slip distributions.

We foresee a need for reappraisal of some other engineering seismology issues as well; such as the impact of incident S-wave azimuth on variability of soil response, the P-wave and S-wave intensity correlations at forward stations used in early warning systems^13^, landslide triggering from seismic actions, risk assessment of spatially extended infrastructures^55^, and ShakeMaps^1^ for rapid responses. Moreover, all of these products rely on ground-shaking maps with spatially cross-correlated predictions, which despite their wide utility^56^ in seismic risk assessment, and reliance on spatial cross-correlation models^57^ derived from residuals of ground-shaking models, have not seen any great revisions in the past few years. Along with the upgrade of existing ground-shaking prediction models, new models from newer and larger datasets^58,59^ providing both Fourier and PSA amplitudes can improve upon our study. Ultimately, the large ground-shaking datasets we have compiled over the last decade are the tools we needed to revisit the old hypotheses, made when data was once scarce.

## Methods

The NGA-West2^2^, KiK-net datasets^3^, and take-off angle tables^38^ used in this study are publicly available. As an electronic supplement, we provide an extract of the KiK-net dataset with all relevant data and model coefficients of equation (2). The peer-reviewed and published isotropic ground-shaking models^33,34^ were developed using either the R-software^60^ or a multi-step mixed-effects regression procedure described in the relevant publications^34^. The equations used to calculate the far-field S-wave radiation coefficients were developed by Aki and Richards^6^.

## Supplementary information

Supplementary Dataset 1
